# G2019s LRRK2 promotes mitochondrial fission and increases TNFα-mediated neuroinflammation responses

**DOI:** 10.1080/19768354.2019.1585948

**Published:** 2019-03-01

**Authors:** Dong Hwan Ho, Heajin Lee, Ilhong Son, Wongi Seol

**Affiliations:** a InAm Neuroscience Research Center, Sanbon Medical Center, College of Medicine, Wonkwang University, Gunpo-si, Republic of Korea; b Electron Microscopy Research Center, Korea Basic Science Institute (KBSI), Daejeon-si, Republic of Korea; c Department of Neurology, Sanbon Medical Center, College of Medicine, Wonkwang University, Gunpo-si, Republic of Korea

**Keywords:** LRRK2, G2019S mutation, Parkisnon’s disease, mitochondria, microglia

## Abstract

Leucine rich-repeat kinase 2 (LRRK2) is involved in the pathogenesis of Parkinson’s disease (PD). LRRK2 has kinase and GTPase activities, and mediates several cell functions, including vesicle trafficking, apoptosis, autophagy, mitochondrial dynamics, and neuroinflammation. G2019S (GS) is the most prevalent mutation of LRRK2. The mutation increases kinase activity, suggesting that this activity is crucial for PD pathogenesis. The activation and inhibition of LRRK2 kinase increases and reduces the levels of proinflammatory cytokines, respectively suggesting that the role of LRRK2 in neuroinflammation is critical for the pathology of PD. Previously, we demonstrated that microglial activation by lipopolysaccharide (LPS) increases mitochondrial fission via the activation of LRRK2 kinase, while LRRK2 kinase inhibition diminishes the fission morphology and release of tumor necrosis factor-alpha (TNFα) in BV2 or rat primary microglia and the brains of GS transgenic mice. In this study, the ectopic expression of GS LRRK2 in BV2 cells significantly elevated the expression of Drp1 along the fragmented mitochondria and decreased mitochondria size compared with controls. GS LRRK2-transfected BV2 cells displayed significantly increased TNFα release and neuronal death. Inhibition of LRRK2 kinase alleviated these features. TNFα levels in brains of GS mice were significantly increased compared to those in their littermates. These data further support our previous findings concerning LPS-induced neuroinflammation and mitochondrial fission in microglia via LRRK2 kinase activation.

## Introduction

Leucine-rich repeat kinase 2 (LRRK2) is associated with Parkinson’s disease (PD) (Khan et al. 2005). LRRK2 harbors kinase and GTPase activity. The regulation between kinase and GTPase activities is a key for the LRRK2-mediated cellular function, which is involved in the pathogenesis of PD (Greggio et al. 2006; Lewis et al. 2007). Recent reports have revealed that the increased LRRK2 kinase activity is important in the dopaminergic loss in the substantia nigra and paracrinergic neuroinflammation in the brain (Heo et al. 2010; Ramonet et al. 2011; Marker et al. 2012; Liu et al. 2015; Puccini et al. 2015; Ho et al. 2017). The stimulations of neuroinflammation and oxidative stress, such as by hydrogen peroxide and rotenone, enhanced LRRK2 kinase activity whereas the inhibition of LRRK2 kinase activity alleviates cell stimulation-induced LRRK2 kinase activity (Dzamko et al. 2012; Yang et al. 2012; Mendivil-Perez et al. 2016; Jang et al. 2018).

Our previous study demonstrated that lipopolysaccharide (LPS)-induced LRRK2 kinase activation mediates neuroinflammation and mitochondrial fission. G2019S (GS) LRRK2 is the most prevalent mutation of LRRK2 (Ho et al. 2018). The resulting hyperactive kinase activity is critical for the pathological initiation of PD. Furthermore, these evidences from LPS- or rotenone-mediated LRRK2 kinase activation in microglia or neuron, respectively, are similar with whole brain analyses, which are containing various cell types including neuron, microglia, and astrocyte, from GS transgenic mice (Ho et al. 2018; Jang et al. 2018). To clarify the neuroinflammatory feature in the microglia of the GS LRRK2 model, we presently transfected BV2 mouse microglia cells with GS LRRK2 to explore several neuroinflammatory responses and paracrinergic features.

## Materials and methods

### Cell culture and transfection

BV2, mouse microglia cells were cultured in high glucose Dulbecco’s modified Eagle’s medium (Cellgro) supplemented with 5% fetal bovine serum (Cellgro) and 1% penicillin–streptomycin (Gibco) in a 5% CO_2_ incubator. The cells were seeded in 35 mm dishes with a coverslip coated by poly-L-lysine (1 × 10^5^) or without coverslip (4 × 10^5^). For GS LRRK2 expression, 1.8 μg of myc tagged-GS LRRK2 plasmid, whose construction has been previously described (Ho et al. 2015), was transfected into BV2 cells using Lipofectamine LTX (Invitrogen). Six hours following transfection, the culture medium was changed and incubation was continued for 36 h with or without GSK2578215A (1 μM, Torcris Bioscience). Cells were fixed with 4% formaldehyde (Wako Pure Chemical Corporation) for immunofluorescence analysis or were harvested for Western blot analysis with 1 × sample buffer.

### Western blot analysis

Harvested samples were sonicated for 10 sec and heated at 65°C for 30 min. Then, samples were loaded onto a 4%–15% gradient pre-cast gel (Bio-Rad Laboratories) for separation of the proteins, which were transferred to nitrocellulose membranes (Amersham). The membranes were exposed to the following primary antibodies: anti-LRRK2 (N241A/34, 75-253, NeuroMabs), anti-Drp1 (C5, 271583, Santa Cruz Biotechnology), anti-Tom20 (FL-145, sc-11415, Santa Cruz Biotechnology), anti-β-actin (sc-47778, Santa Cruz Biotechnology), anti-myc [9E10] (sc-40, Santa Cruz Biotechnology), anti-pS1292 phosphoLRRK2 (MJFR-19-7-8, ab203181, Abcam), anti-α-tubulin (DM1A, T9026, Sigma-Aldrich), anti-TNFα (52B83, sc-52746, Santa Cruz Biotechnology). The secondary antibody was horseradish peroxidase-conjugated goat anti-rabbit or anti-mouse IgG (111-035-003 or 115-035-003, respectively; Jackson ImmunoResearch.). The details have been previously described (Ho et al. 2015).

### Immunofluorescence and quantitative analysis of mitochondria morphology

Fixed BV2 cells were permeablized by 0.1% Triton X-100 in Dulbecco’s phosphate buffered saline (DPBS) for 5 min at room temperature (RT). Coverslips were incubated with a blocking buffer composed of 3% bovine serum albumin and 1% goat serum in DPBS for 1 h. After the blocking step, the mouse monoclonal anti-LRRK2 antibody and rabbit polyclonal anti-Tom20 antibody in blocking buffer were added to cells for 8 h at 4°C followed by the incubation with Alexa Fluoro 488 conjugated anti-mouse (A-11001, Invitrogen) and Texas Red labeled anti-rabbit (T-2767, Invitrogen) secondary antibodies in blocking solution at RT for 2 h. BV2 cells on the inverted coverslip were mounted using ProLong Gold (P36930, Invitrogen) and five images were captured using a Zeiss LS55 confocal microscope operating in the Airyscan mode. BV2 cells expressing GS (*n* = 10) or transfected cells not expressing GS (*n* = 4) were selected and their mitochondria (*n* = 2–20/cell, total 34–107) were analyzed using the Mito-Morphology Macro of Image J software as previously described (Ho et al. 2018).

### TNFα enzyme-linked immunosorbent assay (ELISA) and CCK-8 cell viability assay

Culture media from myc-tagged GS LRRK2 transfected BV2 cells with or without GSK2578215A treatment were collected and concentrated 3-fold using the 3 K filtration tube (Millipore). TNFα was measured using ELISA (Biolegend) according to the manufacturer’s instructions. Each concentrated medium was used to treat SN4741 mouse dopaminergic cells (2 × 10^4^ cells/well) in a 96-well plate for 48 h, and 0.1 μg/ml of the TNFα blocker, Etanercept (Enbrel-Fc, Y BIOLOGICS) was used as a co-treatment. CCK-8 (Dojindo) reagent was added to the SN4741 culture for 30 min, followed by measurement using a Synergy 2 microplate reader (BioTek) at 450 nm.

### Animal care and preparation of brain lysates

GS LRRK2 transgenic mice (Ramonet et al. 2011) were purchased from The Jackson Laboratory (strain B6; C3-Tg [PDGFB-LRRK2*G2019S] 340D jmo/J, stock number 016575). They were housed in a specific pathogen-free facility at the Dankook University Animal Facility. The protocol was approved by the Dankook University Institutional Animal Care and Use Committee (DKU-16-035). Mice were sacrificed by the cervical dislocation. Brains were extracted and lysed in a buffer composed of ice-cold PBS with 1% Triton X-100 and 1 × protease inhibitor cocktail (Calbiochem). Each brain lysate was homogenized using a 17-gauge needle followed by incubation for 30 min at 4°C. After centrifugation at 4,000 × g for 10 min at 4°C, supernatants were transferred to a new tube and subjected to Western blot analysis.

### Data analysis

The protein bands resolved from Western blots were analyzed using a Multi Gauge V3.0 Densitometer (Fuji Film). Data were analyzed using Prism6 (GraphPad software) and are presented by the mean ± standard error of the mean (SEM). Statistical significances are indicated in the figure legends as *p < 0.05, ***p < 0.001, and ****p < 0.0001.

## Results

### Overexpression of G2019S LRRK2 in BV2 promotes mitochondrial fission

**Figure 1.**
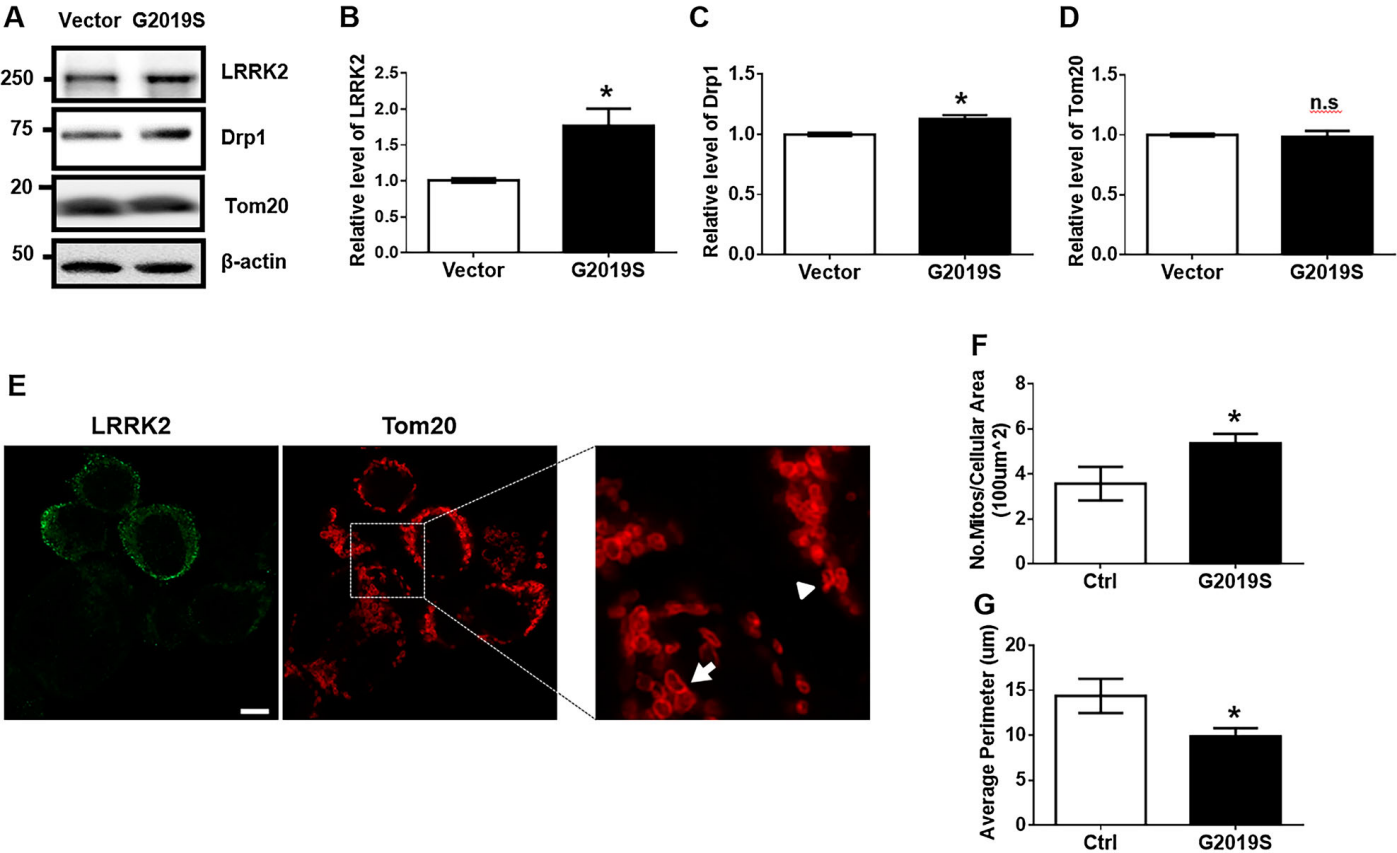
Transfection of G2019S LRRK2 increases mitochondrial fission in BV2. (A-D) BV2 cells were transfected by empty vector (Vector) or myc-GS LRRK2 (GS) using Lipofectamine LTX for 6 h followed by 36 h incubation in the fresh growth medium. Cell lysates were subjected to Western blot analysis. Levels of Drp1 and Tom20 were normalized to α-tubulin levels. (E-G) GS LRRK2-expressing BV2 cells were analyzed by immunofluorescence using anti-LRRK2 [N241A/34] (Alexa-488, green) and anti-Tom20 (Texas Red, red). The morphology of representative mitochondria in GS LRRK2-expressing BV2 (GS) or non-expressing BV2 (Ctrl) is indicated by an arrowhead and an arrow, respectively. Statistical analyses were done using Student’s T*-*test with two-tailed *p*-value; *p < 0.05; n.s., not significant.

Our previous study revealed that LRRK2 kinase activity promotes LPS-mediated mitochondrial fission along with neuroinflammation in BV2 mouse microglia and rat primary microglia (Ho et al. 2018). However, the specific physiological features resulting from GS LRRK2 expression in microglia cells remained elusive. Presently, we transfected BV2 cells with myc-GS LRRK2 and analyzed the mitochondrial fission phenotype using the biochemical and immunofluorescence analyses. GS LRRK2-transfected BV2 (G2019S) displayed a 1.8-fold increase of LRRK2 compared with vector-transfected BV2 (Vector) (Figure 1A, B), indicating that GS transfection led to increased expression of LRRK2. In GS cells, the mitochondrial fission marker, Drp1 protein, was significantly increased, while the mass of the Tom20 mitochondria marker protein was not changed compared with that of the Vector in Western blot analysis (Figure 1A, C, D). Figure 1E presents a representative image of mitochondria from cells expressing GS LRRK2 (G2019S) or non-expressing (Ctrl) indicated by the arrowhead and arrow, respectively. Mitochondria in GS and Ctrl BV2 cells were fragmented and larger, respectively. More mitochondria were evident in the cytoplasm of cells with the GS mutation than in nearby Ctrl BV2 cells (Figure 1F). The mitochondrial perimeter was significantly decreased for GS than for Ctrl (Figure 1G). These results indicated that the role of LRRK2 in LPS-induced mitochondrial fission is dependent on the kinase activity of LRRK2.

### Hyperactive kinase of G2019S LRRK2 provokes TNFα release

**Figure 2.**
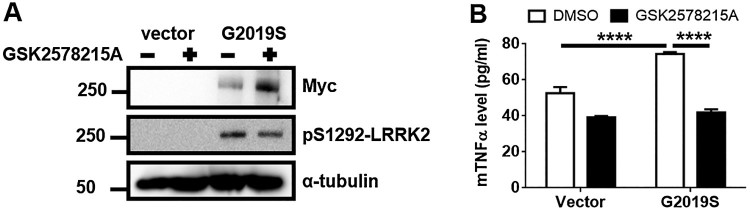
Treatment with LRRK2 kinase inhibitor suppresses the TNFα release in Myc-G2019S LRRK2-expressed BV2 cells. (A) After 6 h transfection, Vector or myc-GS cells were incubated with or without GSK2578215A in the fresh growth medium for 36 h. To validate the LRRK2 transfection and inhibition by GSK2578215A, cell lysates were analyzed using anti-myc and pS1292 antibody. (B) The culture medium was collected and concentrated using a 3 K filtration tube, and the medium was subjected to mouse TNFα ELISA. Two-way analysis of variance (ANOVA) using Tukey’s multiple comparison test was used for the statistical analysis; ****p < 0.0001.

To verify the effect of GS LRRK2 expression on the release of a proinflammatory cytokine, we measured mouse TNFα (mTNFα) levels from GS with or without the treatment of the LRRK2 kinase inhibitor, GSK2578215A. We confirmed the increased phosphorylation of S1292 in LRRK2 from GS lysates (Figure 2A), and the significantly increased level of mTNFα release in GS (Figure 2B). GSK2578215A treatment reduced mTNFα release along with the inhibition of S1292-phosphorylated LRRK2 (Figure 2). The collective data indicated that GS LRRK2 in microglia could be critical for proinflammatory responses.

### Increased secretion of G2019S LRRK2-mediated TNFα increases the neuronal death

**Figure 3.**
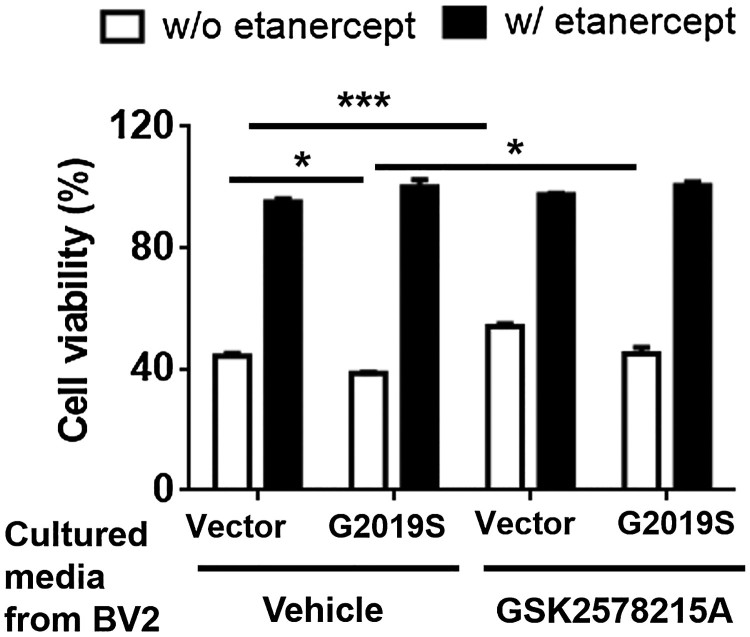
Changes of neuronal cell viability by the cultured media from BV2 cells expressing G2019S LRRK2 with or without the treatment of LRRK2 kinase inhibitor. The collected and concentrated culture medium from the experiment in Figure 2 was use to treat SN4741 cells for 48 h. Etanercept (0.1 µg/ml) was co-treated with concentrated culture medium. The medium was replaced by fresh medium containing 10 µl CCK-8 and incubated for 1 h. Two-way ANOVA of Tukey’s multiple comparison test was performed for the statistical analysis of cell viability; *p < 0.05, ***p < 0.001.

To elucidate the role of GS LRRK2-mediated mTNFα secretion on neurodegeneration, we collected and concentrated the culture media from BV2 cells transfected with GS or Vector with or without treatment of the LRRK2 kinase inhibitor, GSK2578215A. SN4741 mouse dopaminergic neuronal cells were treated with each cultured medium with or without the TNFα blocker etanercept for 48 h. The culture medium from GS displayed significantly decreased neuronal viability compared to the Vector (Figure 3). In contrast, the culture medium from GS treated with GSK2578215A ameliorated neuronal death (Figure 3). Treatment with etanercept rescued all instances of neuronal toxicity (Figure 3). Taken together, the data indicated that TNFα released from GS LRRK2-expressing microglia might mediate neuronal degeneration.

### TNFα levels are increased in brains of G2019S LRRK2 transgenic mice

**Figure 4.**
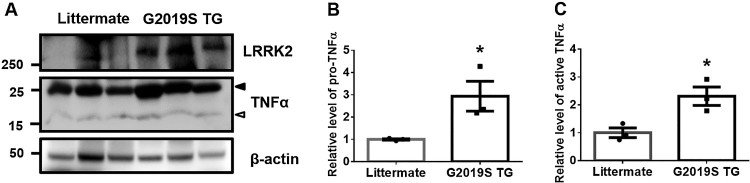
Protein levels of pro- or active-TNFα in brains of G2019S transgenic mice. (A) Brain lysates from littermates and GS TG mice were subjected to Western blot analysis. Arrowhead or empty-arrowhead denotes pro- or active-TNFα, respectively. (B-C) TNFα protein was normalized to the levels of β-actin. Student’s T-test with two-tailed analysis was used for the statistical analysis; *p < 0.05.

To clarify the levels of TNFα in GS LRRK2 transgenic (GS TG) mice, we analyzed the mouse brain lysate. Full-length pro-TNFα, detected a western blot band of approximately 25 kDa, was significantly increased in brain lysates from GS TG mice compared with their littermates (Figure 4A, B). Furthermore, the active form of soluble TNFα, evident as a 15 kDa band, was also significantly increased in GS TG brain lysates (Figure 4A, C). These results suggested that GS LRRK2 plays a critical role in the neuroinflammation, which is a crucial initiator of neurodegeneration.

## Discussion

Previous studies demonstrated that LPS treatment induces mitochondrial fission in BV2 cells (Park et al. 2013; Park et al. 2015). We previously documented that the LPS-induced mitochondrial fission in microglia is mediated by LRRK2 kinase activity (Ho et al. 2018). However, in that study, we had difficulty in observing phenotype changes related to the expression of GS LRRK2 in BV2 cells, probably due to the low transfection efficiency of LRRK2 plasmids in BV2 cells. The LPS-activated microglia would provoke a broad signaling pathway, and LRRK2 is not the only molecule capable of affecting mitochondrial fission. Thus, we needed to optimize ectopic GS LRRK2 expression in BV2 cells to confirm whether LRRK2 kinase could solely promote neuroinflammation and mitochondrial fission. We ultimately succeeded in demonstrating ectopic expression of GS LRRK2 in BV2 cells using a low concentration of DNA for a brief time. Despite the low expression of ectopic LRRK2, we confirmed significant phenotype changes in BV2 cells.

Although the role of GS LRRK2 kinase activity in neuroinflammation is still unclear, various stimuli like LPS as well as Pam3CSK4 for astroglia and rotenone for neurons, have been used to investigate the role of LRRK2 kinase activity in brain cells on the pathogenic mechanism (Dzamko et al. 2012; Mendivil-Perez et al. 2016). Altered mitochondrial dynamics accompanying neuronal degeneration by GS LRRK2 have been reported (Niu et al. 2012; Wang et al. 2012; Saez-Atienzar et al. 2014; Yang S et al. 2014). The link between GS LRRK2-mediated neuroinflammation and neuronal degeneration is not fully understood. Presently, we confirmed that GS LRRK2 can solely mediate mitochondrial fission and the increased TNFα levels in microglia (Figures 1 and 2), thereby aggravating neuronal death (Figure 3). We measured TNFα levels in mouse brain lysates using ELISA. However, result might include both forms of TNFα in the brain (Ho et al. 2018). Both the pro and active forms of TNFα were observed in mouse brain lysates and both forms showed significant changes that were responsible for the neuronal degeneration (Figure 4). Analyses of GS TG mice revealed an increase in mitochondrial fission along with a higher level of CD68, an activated microglia marker, and poly-ADP ribose polymerase, a cell death marker, compared to littermates (Ho et al. 2018). Taken together, the data indicate that the expression of GS LRRK2 in microglia produces neuroinflammatory responses with features similar to those observed in ligand-mediated neuroinflammation. One important thing to note is that the promoter of GS LRRK2 expression in our GS TG mice was a neuron-specific. This prompts the question of how this expression system functions in neuroinflammation. GS LRRK2 expressing neurons increase the secretion of α-synuclein to the extracellular space, which can induce neuroinflammation (Kondo et al. 2011; Bae et al. 2018). This might be a reason for the activated microglial profile in GS TG mice. Investigation of GS LRRK2 expressing microglia using a microglia-specific promotor could confirm the role of LRRK2 kinase activity in the neuroinflammation.

Similar to our previous study that LPS can induce neuroinflammation and mitochondrial fission via LRRK2 kinase activation, we presently observed that the increased LRRK2 kinase activity in microglia cells expressing GS LRRK2 could be responsible for the neuroinflammation along the mitochondrial fission. The data demonstrate that ligand-mediated LRRK2 activation is an easy and helpful surrogate to investigate the role of LRRK2 kinase activity in microglia.
